# Econo-ESA in semantic text similarity

**DOI:** 10.1186/2193-1801-3-149

**Published:** 2014-03-19

**Authors:** Faisal Rahutomo, Masayoshi Aritsugi

**Affiliations:** State Polytechnics of Malang, Soekarno Hatta 9, Malang, Indonesia; Kumamoto University, 2-39-1 Kurokami, Chuo-Ku, Kumamoto, 860-8555 Japan

**Keywords:** Semantic text similarity, ESA, GVSM

## Abstract

Explicit semantic analysis (ESA) utilizes an immense Wikipedia index matrix in its interpreter part. This part of the analysis multiplies a large matrix by a term vector to produce a high-dimensional concept vector. A similarity measurement between two texts is performed between two concept vectors with numerous dimensions. The cost is expensive in both interpretation and similarity measurement steps. This paper proposes an economic scheme of ESA, named econo-ESA. We investigate two aspects of this proposal: dimensional reduction and experiments with various data. We use eight recycling test collections in semantic text similarity. The experimental results show that both the dimensional reduction and test collection characteristics can influence the results. They also show that an appropriate concept reduction of econo-ESA can decrease the cost with minor differences in the results from the original ESA.

**Electronic supplementary material:**

The online version of this article (doi:10.1186/2193-1801-3-149) contains supplementary material, which is available to authorized users.

## Introduction

ESA (Gabrilovich and Markovitch 2007) is a unique approach in information retrieval studies and other related researches. This method measures the relatedness of two texts in a concept space, rather than a term space. In this sense, the relationship is not limited to the lexical form of a text but is expanded to include the meaning. The method uses a straight-forward scenario inside a vector space model. Due to its simple and straight-forward approach, ESA is easy to understand. ESA is actually a variant of a generalized vector space model (GVSM) that uses Wikipedia as its index corpus. Although its theoretical and mathematical foundation is not new, the method is unique because of its use of the constantly growing open-edited online encyclopedia Wikipedia. Wikipedia serves as an additional advantage to ESA because it yields good results for word and text-fragment relatedness measurements (Gabrilovich and Markovitch 2007).

Researchers have further developed ESA for use in different environments, including information retrieval (Hassan and Mihalcea 2009; Polajnar et al. 2013; Potthast et al. 2008; Potthast et al. 2012; Scholl et al. 2010; Sorg and Cimiano 2010; 2012; Tanase and Kapetanios 2012), image retrieval (Popescu and Grefenstette 2011; Zhang et al. 2012), semantic text similarity (STS) (Aggarwal et al. 2012; Martín et al. 2013; Szarvas et al. 2011), categorization (O’Banion et al. 2012; Szarvas et al. 2011; Szczuka et al. 2011), machine translation (Matsuno and Ishida 2011), and question-answering (Walter et al. 2012). It is also used in knowledge discovery (Yan and Jin 2012), music classification (Aryafar and Shokoufandeh 2011), learning systems (Schmidt et al. 2011), text disambiguation (Fernandez et al. 2011), and case based reasoning systems (Patelia et al. 2011). Thanks to Wikipedia’s language links, there are also numerous implementations in multilingual environments (Potthast et al. 2008; Schönhofen et al. 2008; Schmidt et al. 2011; Sorg and Cimiano 2010; 2012; Tanase and Kapetanios 2012).

The method is simple, but the process is expensive because of the following two problems. First, to produce a concept vector, the overall index matrix must be multiplied by a term vector; a large index matrix requires numerous multiplications. Second, if Wikipedia has a million documents, then the concept space has a million dimensions; similarity or relatedness computations between two vectors with numerous dimensions are costly.

Because of these problems, we propose a new scheme called econo-ESA. Econo-ESA reduces the dimensions at the interpretation stage. We call this decrement step a “safe dimensional reduction" because we derive a critical point where the results remain similar despite the reduction. This research provides the following contributions:

– *Runtime reduction*. Econo-ESA introduces a dimensional reduction of ESA in order to decrease the processing runtime while achieving similar results. A reduction in the processing time can be utilized by all relevant areas of research and application because ESA has been used in various applications and environments.

– *Experiments with various data*. We use eight Glasgow test collections with different characteristics in this study. The Empirical evaluation section reports the experimental results in STS. STS measures the similarity between texts based on their meaning. This measurement is closely related to information retrieval, text disambiguation, and machine translation, where STS can be used as a base. The results allow us to set the amount of dimensional reduction according to our own applications.

Before outlining our method, we briefly explain ESA/GVSM in Section ‘ESA/GVSM overview’. We discuss related improvements to the method in Section ‘Improvements to the method’ and our proposed position toward each improvement. Section ‘Econo-ESA’ outlines our proposal, econo-ESA, in detail. Section ‘Empirical evaluation’ provides the experimental results and discussions of our proposed method and the original scheme. Section ‘Conclusion’ offers conclusions and recommendations for future study.

## ESA/GVSM overview

This section briefly explains ESA/GVSM in order to provide a fundamental overview of this method as it relates to the overall discussion in this paper. The mathematical operation of ESA underlies the pseudo-code of the procedure in Section ‘Econo-ESA’. A procedural analysis then evaluates the superiority of our method in Section ‘Empirical evaluation’.

ESA (Gabrilovich and Markovitch 2007) is a variant of GVSM (Wong et al. 1987) and can be considered a Wikipedia-based GVSM (Anderka and Stein 2009). ESA considers each Wikipedia document to hold a unique concept that is described by its text. The preprocessing of an overall Wikipedia corpus produces a matrix **I**. Document numbers *m* and vocabulary terms *n* of Wikipedia define the size of the matrix as *m*×*n*. This matrix is typically read as the term vectors of the documents. ESA transposes the matrix as **I**^⊤^ with size *n*×*m* and then defines it as a concept vector of terms. This **I**^⊤^ matrix is the index matrix of the ESA, the heart of this method.

ESA interprets a term vector **x** (with *n* dimensions) as a Wikipedia-based concept vector **v** (with *m* dimensions) by multiplying the index matrix **I**^⊤^ by the term vector **x**. This multiplication represents a term vector of a text to a higher vector space that is considered to be a concept space.

Each weight *v*_*j*_ of concept dimension *v*_*j*_ in vector **v** is defined as (Gabrilovich and Markovitch 2007): 1

where *x*_*k*_ is the dimension weight of term *i* in vector **x**, and *i*_*jk*_ is the weight of concept *j* for term *i*. The weight of a term or a concept can be determined by its term frequency, collection frequency, or normalization component (Salton and Buckley 1988).

If the ESA measures the semantic relatedness of two texts, then both texts are represented into two concept vectors **u** and **v**. The measurement of the vectors can then be accomplished in the vector space of the concept by a vector measurement such as cosine similarity: 2

This concept is the same as in GVSM. GVSM allows a transformed vector **x**^′^ of vector **x** to be generated with this function (Yang et al. 1998): 3

where **A**^⊤^ is an index matrix that is derived from a corpus.

Based on the above explanation, ESA computes the similarity of two texts through two stages: an interpretation and a similarity measurement stage. We propose reducing the index matrix **I**^⊤^/ **A**^⊤^ by reducing the number of documents, thus reducing the dimensions of concept space. This proposal requires less multiplication and thus achieves faster processing time in both stages of ESA.

## Improvements to the method

This section describes the position of our proposal as compared to other research and discusses related improvements to the method in detail. We compare our proposal to each improvement and summarize related studies based on Sorg and Cimiano’s framework (Sorg and Cimiano 2010). This framework considers the index matrix, association strength, similarity measurement, and dimension limitation as ESA enhancements. We also consider the semantic interpretation process as an additional aspect to the framework. The prior enhancements are described below.

### Index matrix

ESA uses a Wikipedia corpus as its index matrix source. ESA also tests an ODP corpus and compares the results with Wikipedia. Hassan and Mihalcea (2009) used Wikipedia’s graph of category and intra-lingual links; they used the category to scale a concept’s weight based on its distance to the root and its intra-lingual link to map a concept into the other languages. Martín et al. (2013) used a collection of research papers as an ESA corpus. Potthast et al. (2008) proposed a cross-language ESA (CL-ESA) for cross-language similarity measurements. They defined an independent concept space that referred to the different language of the Wikipedia corpus. Sorg and Cimiano (2012) proposed a Cat-ESA and a Tree-ESA that deployed the category structures of Wikipedia. Szarvas et al. (2011) reported experimental results on the German and English knowledge resources of Wikipedia, Wiktionary, and WordNet/GermaNet for combination concept vector measurements. Aryafar and Shokoufandeh (2011) proposed using a music collection as an index matrix. Popescu and Grefenstette (2011) proposed using categories instead of Wikipedia articles. Szczuka et al. (2011) used Dbpedia. Gottron et al. (2011) proposed an index matrix with the same topic as the test collection to provide a better result. Scholl et al. (2010) proposed an extended ESA that used other Wikipedia semantic properties such as article links and categories. Polajnar et al. (2013) proposed a new index matrix semantic kernel of *n*×*n* size, thus describing concept-to-concept similarity.

Although econo-ESA works in an index matrix such as (Aryafar and Shokoufandeh 2011; Gottron et al. 2011; Hassan and Mihalcea 2009; Martín et al. 2013; Polajnar et al. 2013; Popescu and Grefenstette 2011; Potthast et al. 2008; Scholl et al. 2010; Sorg and Cimiano 2012; Szczuka et al. 2011; Szarvas et al. 2011), we do not propose a new index matrix. We intend to investigate the dimensions of the matrix by showing that econo-ESA decreases the index matrix’s dimensions appropriately.

### Association strength

An association strength function derives the values of each cell within an ESA index matrix. The original ESA uses a TFIDF scheme. Hassan and Mihalcea (2009) refined ESA association strength by considering the length of the articles. Sorg and Cimiano (2010) examined five association strength schemes: TF, TFIDF, TFIDF*, BM25, and cosine. They (Sorg and Cimiano 2012) also introduced TFICF, TFICF^2^, and TFICF^3^. TFICF provides the same function as TFIDF but for a concept space, while TFICF^2^ and TFICF^3^ implement quadratic and cubic values for ICF, respectively. Aryafar and Shokoufandeh (2011) used a mel-frequency cepstral coefficient (MFCC) scheme for their music index matrix. Fernandez et al. (2011) proposed a balanced ESA that considered the differences between short and long articles in Wikipedia.

We do not propose a new association strength scheme such as (Aryafar and Shokoufandeh 2011; Fernandez et al. 2011; Hassan and Mihalcea 2009; Sorg and Cimiano 2010; 2012). Econo-ESA uses an original ESA scheme, TFIDF, to highlight the improvement of our proposal to the original ESA.

### Similarity measurement

ESA utilizes a cosine metric to compute the semantic relatedness of two concept vectors. Hassan and Mihalcea (2009) replaced cosine similarity with a Lesk-like metric. Martín et al. (2013) explored two similarity measurements of two concept vectors: a cosine and a generalized jaccard. Sorg and Cimiano (2010) explored the TFIDF, KL-divergence, and LM schemes in addition to cosine similarity. Aggarwal et al. (2012) proposed TunedESA, an ESA with an added tuning feature. This method tunes the ESA with three WordNet-based Lin semantic similarities of word functions in two sentences — the subject, action, and object of each sentence. Yan and Jin (2012) integrated the TFIDF similarity score with the ESA-based similarity score. Szarvas et al. (2011) proposed a new similarity measurement: . The relatedness of terms *t*_1_ and *t*_2_ is determined by the term’s concept vector weight,  and .

The econo-ESA uses a cosine measurement (Equation (2)) for the similarity measurement (same as the ESA) in order to directly evaluate our proposal by comparing it to the original ESA. Although it is obviously easy to use other schemes in our proposal, we do not propose a new similarity scheme such as (Aggarwal et al. 2012; Hassan and Mihalcea 2009; Martín et al. 2013; Sorg and Cimiano 2010; Szarvas et al. 2011; Yan and Jin 2012).

### Semantic interpretation process

To produce a concept vector, the original ESA multiplies a vector text and an index matrix. Martín et al. (2013) proposed a new method of semantic interpretation that considered the keywords, author, or journal information of a corpus. During the interpretation process, Potthast et al. (2012) proposed an ESA _△_ that reduced terms in text *d* that appeared in text *d*_*q*_, thus forming a new text *d*_△_. The similarity between *d* and *d*_*q*_ was measured by the similarity between *d*_△_ and *d*_*q*_. Schmidt et al. (2011) proposed two mapping scenarios – direct CL and meta CL. Direct CL uses Wikipedia’s inter-language link if it exists; if the link does not exist, then the method uses meta CL mapping.

Econo-ESA uses an original scheme of the semantic interpretation process. We do not propose a new strategy such as (Martín et al. 2013; Potthast et al. 2012; Schmidt et al. 2011).

### Dimension limitation

Limitations to the dimension come under several proposals: a dimension projection function, dimensional pruning, and the number of index documents. We will describe each proposal idea below and outline the difference of econo-ESA as compared to these proposals.

#### Dimension projection function

Sorg and Cimiano (2010) formalized the limitation and proposed four dimension limitation functions: absolute, absolute threshold, relative threshold, and sliding window. All functions work in the concept space. The absolute function limits the dimension to *d* dimensions with the highest value. The absolute threshold function limits the dimensions based on their values toward a threshold. The relative threshold function uses a partial fraction of the highest-valued dimension. The sliding window function selects the first *i* dimensions from the results of the relative threshold function. All the above dimension projection functions are performed according to the ordered vector concept. As noted in Section ‘ESA/GVSM overview’, this process occurs after the interpretation process.

#### Dimensional pruning

Sorg and Cimiano (2012) also proposed dimensional pruning. Dimensions with values under a specified threshold are set to zero. Similar to the dimension projection function, this step is performed after the interpretation step.

#### Number of index documents

Anderka and Stein (2009) showed that a larger index matrix size (i.e., greater than 200,000 documents) provided more stable results. They also show that additional documents in the index matrix increased the stability. However, their experiments used a test collection of only 50 documents from the Australian Broadcasting Corporation’s news mail service.

#### Dimension limitation of econo-ESA

To reduce the processing cost, econo-ESA modifies the index matrix of ESA and reduces the number of dimensions. This study is similar to previous proposals (Anderka and Stein 2009; Sorg and Cimiano 2010; 2012) but additionally considers the cost reduction of the processes. Econo-ESA reduces costs by limiting the dimensions at the interpretation stage, and that is different from previous studies. Because of this, the overall processing cost can be decreased in both the interpretation and relatedness measurement stages. Previous proposals decreased computational costs in the last step, relatedness measurement, but did not decrease the interpretation step costs. This required the interpretation step to be performed on the entire index matrix of the system. Moreover, an additional vector sorting step (Sorg and Cimiano 2010; 2012) required before the reduction also adds to the cost. We used eight test sets with different characteristics, while (Anderka and Stein 2009) performed experiments with a single test collection. The goal is to identify the trade-off between dimension reduction, processing time, and performance while considering whether test collection characteristics may affect the results. Our experimental results can be used to guide the selection of appropriate reductions in accordance with the actual conditions as required. Based on these results, the reader can determine the proper reduction based on desired performance and speed, as well as the characteristics of the text to be processed; the results of (Anderka and Stein 2009) cannot be used for this purpose.

## Econo-ESA

This section describes an economics-based ESA named econo-ESA that proposes a new research direction: decreasing ESA costs with negligible impact to the results. This research focuses on the index matrix and the limitation of the dimension. Therefore, we use original ESA schemes in the following aspects: association strengths, similarity measurements, and semantic interpretation processes.

The subsections that follow explain the concept of econo-ESA. The safe dimensional reduction decreases the cost with negligible impact to the results. The procedural analysis then explains how a faster procedure can be achieved by the decrement.

### Safe dimensional reduction

The econo-ESA proposal suggests a way to determine how much the index matrix can be reduced without affecting the results. To our knowledge, this proposal has not been suggested in previous studies (Aggarwal et al. 2012; Anderka and Stein 2009; Aryafar and Shokoufandeh 2011; Fernandez et al. 2011; Gottron et al. 2011; Hassan and Mihalcea 2009; Martín et al. 2013; Polajnar et al. 2013; Popescu and Grefenstette 2011; Potthast et al. 2008; Scholl et al. 2010; Sorg and Cimiano 2010; 2012; Szczuka et al. 2011; Szarvas et al. 2011; Yan and Jin 2012). We propose a dimensional reduction of the Wikipedia matrix. The proposal refers to a previous ESA experiment with different index matrix sizes, as shown in (Anderka and Stein 2009). Based on these data, we can build a fit model with an appropriate regression technique. After deriving the model from the data, we select one data point with a least squared error value. This point refers to the number of documents that have a percentage amount of the total corpus.

Table 1 shows the ESA experimental data of Pearson’s correlation coefficient (*PCC*) with different index matrix conditions. The *PCC* of two data sets *x* and *y* is determined by Equation (4). The experiments were performed for 1,000, 10,000, 50,000, 100,000, 150,000, and 200,000 random Wikipedia documents. The experiments were performed for several association strength schemes for several corpora. Table 1 shows one row of data resulting from the Wikipedia TFIDF index matrix. We chose this row because it is an original ESA scheme. 4

**Table 1:** ***PCC*** **of ESA based on the number of index documents (** Anderka and Stein 2009 **)**

Index collection	Number of index documents					
	1,000	10,000	50,000	100,000	150,000	200,000
Wikipedia TFIDF	0.742	0.784	0.782	0.782	0.781	0.781

Based on Table 1, we then built a model of ESA *PCC* performance. We chose two nonlinear regression models: logarithmic and logistic. The curves of both models apparently fit the data shown in Table 1. The logarithmic and logistic model functions of document numbers *x* and *PCC* prediction *PCCpr* are shown in Equations (5) and (6), respectively (Walpole et al. 2007). 5

6

We used OpenOffice Calc solver and MS Office Excel logarithmic trendline to define the logarithmic function’s variable value. Calc produced *β*_0_ and *β*_1_ as 0.706869 and 0.006565, respectively. Excel produced *β*_0_ and *β*_1_ as 0.706 and 0.006, respectively. We used the logistic regression procedure of R statistic software for the logistic model. The software gave results for *b*_0_ and *b*_1_ as 1.192 and 5.541×10^−7^, respectively.

**Figure 1:**
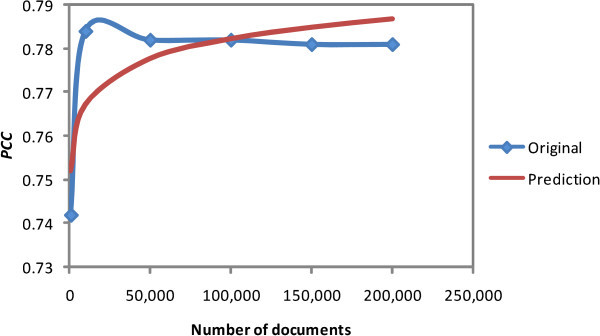
**Logarithmic model.**

We selected one model from the three based on the model’s mean squared error (MSE) (Additional file 1 with yellow highlight). The least MSE value from the three is the logarithmic model generated by OpenOffice Calc solver, as shown in Table 2. We then selected the model generated by Calc for this econo-ESA proposal, as shown in Figure 1.

**Table 2:** **MSE comparison**

Model	MSE
Logarithmic OpenOffice solver	7.53483E-05
Logarithmic MS excel trendline	0.000122114
Logistic R	0.000174

Now we determine the econo-ESA decremental percentage. From the model shown in Figure 1, we consider a point with the least squared error value between the model and the data (Additional file 1 with blue highlight). The requirement conformed with 100,000 documents, half of the 200,000 documents in the experiment. The *PCC* and *PCC*_*pr*_ of this point are 0.782 and 0.782448, respectively. The similarity between the two values is 0.999427 with Equation (7). 7

Based on this fact, we develop our expectation: Econo-ESA is similar to ESA at a 50% reduction of the dimensions. Our experiments are reported in the Empirical evaluation section.

### Procedural analysis

This subsection describes the cost savings of econo-ESA. We consider a common procedure of ESA in two parts: the interpretation process procedure and the cosine similarity procedure. We then analyze the processing costs.

#### Interpretation procedure

Procedure 1 is an implementation of the ESA interpretation step. Different implementations may exist based on each implementer choice. The procedural input is an array *T* of a term vector and a matrix *W* of a Wikipedia corpus. The array has *n* elements based on the vocabulary term amount in the system. Matrix *W* keeps Wikipedia’s inverted index *m*×*n* for the interpretation process. The output of this procedure is an array *C* with *m* elements that matches the total number of documents in the corpus. An element of array *C* indicates the weight of concept dimension in the dimension space.

##### **Procedure 1** : Interpret a given term vector into a concept vector



According to Procedure 1, the computational cost *C*_*I*_ is: 

If we decrease the number of documents to 50% of *m*, the value of *C*_*I*_ is halved; this means that the procedure becomes twice as fast. We expect to compute ESA with similar results. Variable *n* also appears in the above analysis. This result shows that more terms present in a text require more running time.

#### Cosine similarity

Procedure 2 is a cosine similarity implementation. The procedural input consists of *C*_1_ and *C*_2_ arrays that represent concept vectors **c**_**1**_ and **c**_**2**_. The output of this procedure is the similarity value, a fractional number between 0 and 1.

##### **Procedure 2** : Measure similarity score of two concept vectors



The analysis of Procedure 2 shows that document number *m* also affects the cost *C*_*M*_ in the similarity computation step as follows: 

Similar to Procedure 1, if we decrease the number of documents by 50% of *m*, then the value of *C*_*M*_ is halved. This further strengthens the advantage of the econo-ESA proposal.

#### Running cost of typical STS computation

One STS computation by ESA requires two interpretation steps and one cosine similarity step. The overall running cost can be defined as: 8

If we reduce *m* to 50% of *m*, the overall cost is halved, meaning that the procedure will be twice as fast.

## Empirical evaluation

### Experimental setup

To evaluate the superiority of our proposal to the original ESA, widely used techniques are adopted. We evaluate the *PCC*, precision, recall, F-score, and running time of the ESA and econo-ESA systems. *PCC*, precision, recall, and F-score evaluate the similarity of both systems, while running time evaluates the costs.

We used the Microsoft Wikipedia corpus (MSwik) from (Yih et al. 2011) in the semantic interpreter part of the ESA. The MSwik size is 218.44 MB and can be downloaded from http://research.microsoft.com/en-us/downloads/fd8811ca-a57f-4803-8f5c-41b536bf3a80/. This data set consists of 60,730 samples of Wikipedia English-Spanish articles from 2009. We only used the English articles from this data set, consisting of 20,000 terms. ESA used all 60,730 documents, while econo-ESA used only half—30,364 documents. We also ran 40%, 60%, and 70% of econo-ESA for comparative purposes, with 24,292, 36,438, and 42,511 documents, respectively. We randomly selected the documents. We used terms "econo40", "econo50", "econo60", and "econo70" for each econo-ESA scheme based on their semantic interpreter size. The ESA and econo-ESA implementations were performed in Perl 5.12.3 and MySQL 5.5.16. Experiments ran on a 3.4GHz Intel COREi7 PC with 8GB RAM.

For evaluation purposes, we used eight recycling test collections generated from Glasgow test collections. All test sets can be accessed from http://ir.dcs.gla.ac.uk/resources/test_collections/. These eight test collections were fitted to the semantic test similarity task as reported in (Rahutomo et al. 2012). We used these test collections because of their characteristic variations, which are shown in Table 3. “Docs.”, “Qys.”, and “Rel.” columns describe each dataset number of documents, queries, and relevant pairs, respectively. The next columns show term distribution characteristics of document texts and query texts. “Min.”, “Q1”, “Med.”, “Q3”, and “Max.” sub-columns describe the minimum, first quarter, median, third quarter, and maximum values, respectively. We pre-processed all eight Glasgow test collections as if they were MSwik. We referred to the 20,000 term library in MSwik. The pre-processing of the texts applied neither stemming nor folding to the terms and determined their TFIDF values. The ESA and econo-ESA then transformed each document or query term vector into a concept vector. Finally, the cosine similarity metric measured the semantic similarity between the document and query concept vectors.

**Table 3:** **Test collection characteristics**

Dataset	Docs.	Qys.	Rel.	Document terms				
				Min.	Q1	Med.	Q3	Max.
LISA	6,004	35	335	11	68	96	128.25	352
NPL	11,429	93	2,083	3	25	39	58	293
CACM	3,204	64	796	3	10	23	108	455
CISI	1,460	112	3,114	13	97	137	186	676
Cranfield	1,400	225	1,838	1	113	165	241.25	738
Time	423	83	324	91	399	612	918	6,618
Medline	1,033	30	696	24	107	159	226	758
ADI	82	35	170	28	60.25	70.5	80	216
**Query terms**					**Explanation**			
**Min.**	**Q1**	**Med.**	**Q3**	**Max.**				
23	49.5	64	85	142	Abstracts collection			
4	9	12	15	24	Short text			
3	8.75	16	30	62	CACM articles index			
4	20	72	122.75	335	Index of articles			
6	12	16	21	43	Index of articles			
8	15	20	23.5	46	Short text			
3	9.25	16.5	23.75	60	Medical text			
4	8	13	21.5	57	Short articles			

Because the test collections were derived from the information retrieval data set, human judgment forms are expressed in binary value. To measure *PCC*, we selected two similarity thresholds for both the ESA and econo-ESA schemes — 0.5 and 0.6 — based on previous study (Islam and Inkpen 2008; Mihalcea et al. 2006) constraints.

We then measured the running time for the processing cost. We used the special tag “:hireswallclock” for a benchmark package in our Perl application. We measured the running time of several processes: interpretation, cosine measurement, and STS. For the interpretation process, we measured the running time of the document and query texts for both ESA and econo-ESA. We chose five randomly selected texts from all of the test collections. For the cosine similarity procedure, we measured the running time of five randomly selected documents and query pairs from all of the test sets. For the STS process, we combined the previous results of the interpretation and cosine measurement processes. We then calculated and compared the average scores.

### Results and discussion

#### ESA and econo-ESA correlation

Tables 4 and 5 show the *PCC* evaluation results for the ESA and econo-ESA schemes with 0.5 and 0.6 similarity thresholds, respectively. The experimental results show that econo-ESA achieved our first expectation. The econo-ESA results are similar to those of ESA at a 50% decremental of documents. The average *PCC* values for the similarity thresholds of 0.5 and 0.6 were 0.9226 and 0.8276, respectively. The best result is identical between ESA and econo-ESA, at the 0.5 similarity threshold, while the worst at the 0.6 similarity threshold. A further inspection of the tables show that the variation in test collections influenced the results, whether in collection size or term size of the text.

**Table 4:** ***PCC*** **of 0.5 similarity threshold**

Test set	***PCC***			
	econo40	econo50	econo60	econo70
LISA	0.8332	0.8381	0.8486	0.8610
NPL	0.9785	0.9820	0.9849	0.9867
CACM	0.8053	0.8469	0.8615	0.9084
CISI	0.8825	0.8929	0.9062	0.9164
Cranfield	0.8756	0.9798	0.9153	0.9254
Time	0.8498	0.8716	0.8797	0.8950
Medline	0.8793	0.9693	0.8737	0.8820
ADI	1.0000	1.0000	1.0000	1.0000
Average	0.8880	0.9226	0.9087	0.9219

**Table 5:** ***PCC*** **of 0.6 similarity threshold**

Test set	***PCC***			
	econo40	econo50	econo60	econo70
LISA	0.8510	0.8670	0.8853	0.8942
NPL	0.9508	0.9656	0.9782	0.9827
CACM	0.7322	0.7709	0.8212	0.8776
CISI	0.8446	0.8611	0.8772	0.8909
Cranfield	0.7913	0.9443	0.8617	0.8746
Time	0.7875	0.8257	0.8494	0.8669
Medline	0.7727	0.9505	0.7829	0.7885
ADI	0.4357	0.4357	0.5901	0.6127
Average	0.7707	0.8276	0.8307	0.8485

We present the results differently from those of Anderka and Stein (2009) for several reasons. First, their results were gathered from the *PCC* of ESA and human judgment results. These judgments came from eight to twelve individual judgments. The averages of these judgments become human judgment values, a fractional number between 0 and 1. We do not follow this approach because human judgments in the Glasgow test collections (the test collections we used) are binary values of either 0 or 1. Second, there are no *PCC* values between the results of the ESA with different index matrices in Table 1; we therefore cannot determine these values because we do not have complete raw results of the experiments. We cannot determine the  value of Equation (4). Because of this, we do not compare our results to those of Anderka and Stein, but we do compare the ESA and econo-ESA results.

In addition, the collection size affects the row amount of the experiment. Similarity computations were performed for all possible pairs of document and query texts. The incremental threshold value decreased the *PCC* values of all test sets except LISA. The decrements were between 0.0164 and 0.5643. The decrement in the *PCC* of ADI was the highest because ADI had the fewest test collections. ADI had only 82 documents and 35 queries, thus producing 2,870 rows. Small differences between ESA and econo-ESA results yielded a greater *PCC* decrement. Contrary to ADI, NPL had the greatest number of collections, with 11,429 documents and 93 queries. Robust test collection is more stable in threshold tuning, as seen in NPL, while minimal collection is more susceptible, as seen in ADI.

As for term size, we cannot discern any pattern from the results. The term sizes of NPL and CACM are small, but the results are very different. NPL’s *PCC* is the highest among the eight test sets, but CACM’s *PCC* is the second lowest. Cranfield and Medline have a large term size along with their high *PCC*, which is more than 0.9. Time has a greater term size than Cranfield and Medline, but its *PCC* is lower than these two. NPL has a smaller term size but a higher *PCC* than these two. There is no impact of term size in each test collection based on their *PCC*. According to our experiments, a 50% decrementation of the Wikipedia index matrix yields similar results for different test collections.

The other econo-ESA schemes show that the increase in *PCC* is directly proportional to the number of documents used in the interpretation. In Table 4, under the *PCC* of 0.5 similarity threshold, all of the test sets show this trend except for Cranfield and Medline. For both test sets, econo50 is superior to all other schemes. The average value of econo50 is also superior to the other schemes according to the Cranfield and Medline results. In Table 5, at the *PCC* of 0.6 similarity threshold, the results are similar to the Table 4 trends. Econo50 is superior for the other schemes for Cranfield and Medline test sets. However, Table 5 shows different average results from Table 4. In Table 5, econo70 is the best scheme.

#### ESA and econo-ESA STS performance

Tables 6 and 7 show the precision, recall, and F-score performance of both ESA and econo-ESA schemes. Tables 6 and 7 also provide the results based on the 0.5 and 0.6 similarity thresholds, respectively. Table 6 shows the same precision results in the LISA, NPL, CACM, CISI, Medline, and ADI experiments. Table 7 shows the same facts in the LISA, NPL, CACM, CISI, and Cranfield experiments. Most of the F-score values between ESA and econo-ESA are the same. Although the results are different, the gaps are small. Distinctive results are found in all of the recall metric experiments except for ADI at the 0.5 similarity threshold. Nevertheless, the differences are small. Econo-ESA at the 50% decremental of the ESA index matrix shows similar results to the ESA.

**Table 6:** **Precision, Recall, and F-score of ESA and econo-ESA with 0.5 threshold value**

Test set	ESA			Econo-ESA		
	Precision	Recall	F-score	Precision	Recall	F-score
LISA	0.0018	1.0000	0.0036	0.0018	0.9983	0.0036
NPL	0.0020	0.9884	0.0039	0.0020	0.9905	0.0039
CACM	0.0048	0.9922	0.0095	0.0048	0.9915	0.0095
CISI	0.0280	0.9700	0.0533	0.0280	0.9637	0.0532
Cranfield	0.0060	0.9614	0.0118	0.0059	0.9617	0.0118
Time	0.0094	0.8457	0.0184	0.0092	0.7790	0.0180
Medline	0.0224	0.9650	0.0436	0.0224	0.9636	0.0435
ADI	0.0590	0.9905	0.1054	0.0590	0.9905	0.1054

**Table 7:** **Precision, Recall, and F-score of ESA and econo-ESA with 0.6 threshold value**

Test set	ESA			Econo-ESA		
	Precision	Recall	F-score	Precision	Recall	F-score
LISA	0.0018	0.9875	0.0036	0.0018	0.9863	0.0036
NPL	0.0020	0.9767	0.0039	0.0020	0.9770	0.0039
CACM	0.0049	0.9692	0.0097	0.0049	0.9724	0.0097
CISI	0.0279	0.9162	0.0531	0.0280	0.8912	0.0531
Cranfield	0.0060	0.8885	0.0119	0.0060	0.9008	0.0120
Time	0.0100	0.6742	0.0194	0.0103	0.6179	0.0198
Medline	0.0228	0.8500	0.0440	0.0227	0.8600	0.0440
ADI	0.0597	0.9808	0.1062	0.0592	0.9905	0.1059

#### Processing cost

**Figure 2:**
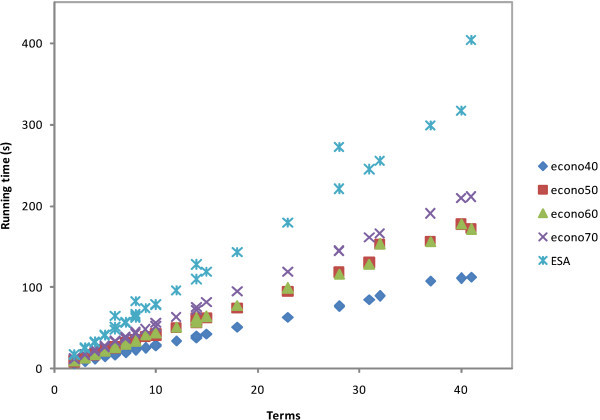
**Running time of query texts.**

**Figure 3:**
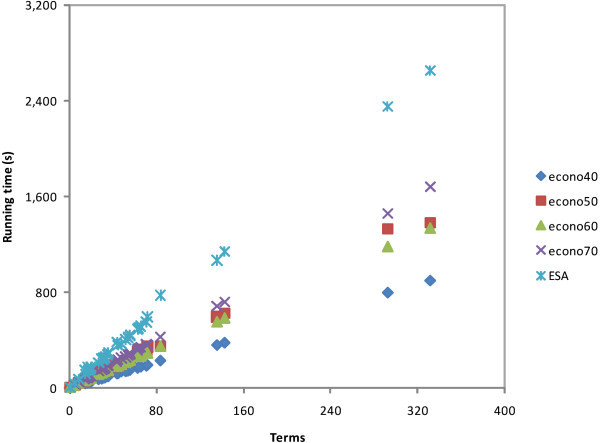
**Running time of document texts.**

**Figure 4:**
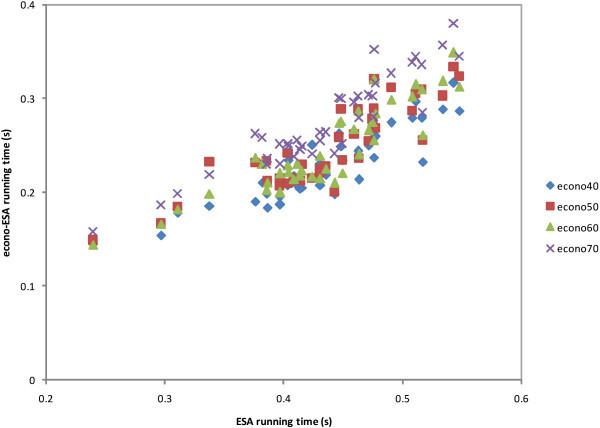
**ESA/econo-ESA scatter plot of cosine similarity procedure.**

**Figure 5:**
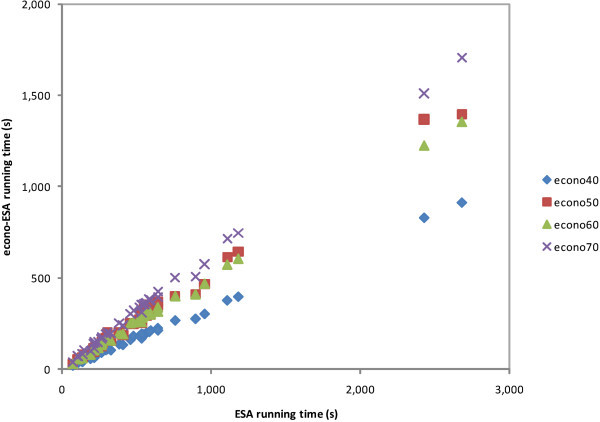
**ESA/econo-ESA scatter plot of STS procedure.**

**Figure 6:**
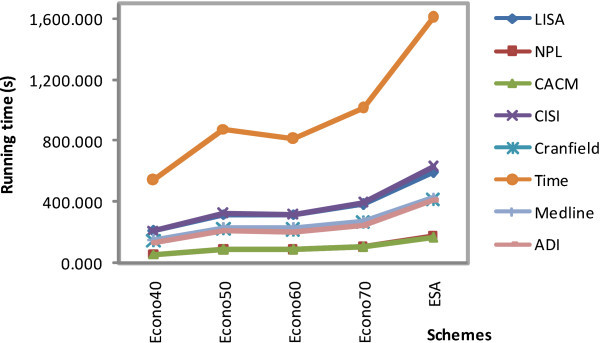
**Running time of the different schemes.**

The experimental results are shown in Figures 2, 3, 4, 5, and 6. Interpretation experiments were performed for five random queries and documents of all test collections. Figure 2 shows the interpretation cost diagram for all query experiments. We calculated the ratio between the ESA and econo-ESA schemes and retained their averages in the first row of Table 8. As shown in the results, econo40 was the fastest scheme, followed by econo50, econo60, and econo70. The document number of the index matrix clearly influences the results.

**Table 8:** **ESA/econo-ESA cost ratio**

Process	ESA/econo40	ESA/econo50	ESA/econo60	ESA/econo70
Query	2.9011	1.9538	1.9113	1.5453
Document	2.8703	2.0141	1.9470	1.5842
Cosine	1.8950	1.7408	1.7908	1.5949
STS	2.9230	1.9998	1.9611	1.5925

Figure 3 shows the interpretation costs of all the documents. The cost ratios between ESA and econo-ESA are shown in the second row of Table 8. The trend is the same as that in the previous process; in row 2, the running cost increases while the Wikipedia corpus is decreased.

We measured the cosine similarity processing cost of five pairs from each test collection. This experiment collected 40 measurement data of all test collections of all schemes. The results are shown in Figure 4, which shows a running time scatter plot comparison between ESA and econo-ESA for the same text pairs. The average values of the ESA and econo-ESA ratios are shown in the third row of Table 8. In this process, econo60 is faster than econo50. Note, however, that we only used 40 measurement data from randomly selected texts in the experiment; we may have slightly different results if we used more texts. In fact, the gap between econo50 and econo60 is very small. A further inspection of the results shows that we cannot find any pattern based upon the number of terms; econo50 and econo60 randomly beat each other. Based on this fact, we can also consider that the 60% index matrix of econo-ESA is also a good candidate because the *PCC* of econo60 is better than econo50 at the 0.6 similarity threshold. The term amount of a text has no impact on this process because each term vector of text has been interpreted as a concept vector with the same dimension.

Figure 5 shows the running time scatter plot of typical STS processing cost. The figure shows 40 experimental data comparisons between ESA and econo-ESA for the same text pairs of all test collections. The measurements were performed for all schemes. We obtained the running cost from the results of previous interpretation and cosine measurement experiments. The fourth row of Table 8 shows the experimental averages.

In general, the additional document of the index matrix increases the processing cost, as shown in Figure 6. Close to the calculations in the Procedural analysis section, econo50 is twice as fast as the original ESA. However, based on our experiments, econo60 is faster than econo50 in the cosine similarity process. In a typical STS process, which includes the interpretation portion of a query, document, and a cosine similarity process, the number of terms influences the result. As shown in Figure 6, Time is the most costly among the test sets because of its term size. In these experiments, econo50 achieved our expectation. We can also consider econo60 as our next econo-ESA candidate because it has shown better results than econo50 in several instances.

#### Results trade-off and further use

We examined the experimental results in terms of processing time, precision, recall, and F-score, which were not shown in the previous study (Anderka and Stein 2009). Therefore, we can provide an overview analysis of the trade-offs. In general, if we decrease the index matrix, the processes will run faster but the correlation with original ESA will decrease. Precision, recall, and F-score between ESA and econo50 are the same or very close. We determined that the 50% index matrix reduction is the best based on the analysis in Section ‘Safe dimensional reduction’, but we also found that the results were not always the same as our expectation. Econo50 is superior for Cranfield and Medline in both the 0.5 and 0.6 similarity thresholds, but not for the other test sets. As for the processing time, we found that the cost ratio of ESA/econo-ESA between econo50 and econo60 do not differ much in any processes. Therefore, we can use econo60 for better results than econo50 with little extra cost.

We hoped to obtain a pattern based on the test set characteristics. While we determined that a small test set such as ADI will be strongly impacted by the change in the similarity threshold, we could not show a clear relationship between the amount of dimension reduction, the amount of text in the test set, and the *PCC* in the results, particularly with Cranfield and Medline. We intend to investigate how we can balance these relationships in the future.

The results indicate that econo50 provides good results. A slight cost sacrifice to econo60 can improve outcomes over econo50. Econo60 usage may be considered for short texts because it has a ramp slope of running time similar to CACM and NPL, as shown in Figure 6. For longer texts such as Time, the running time covers a steep slope, so the use of econo50 is advised.

## Conclusion

This paper proposed an econo-ESA scheme. This method decreases the cost of ESA while providing similar results. We proposed a decremental method as a safe dimensional reduction that uses modelling steps to determine an appropriate decremental percentage of 50%. Our experiment showed that a 50% decrement of the index matrix had average *PCC* results of 0.9226 and 0.8276 for the 0.5 and 0.6 similarity thresholds, respectively. Precision, recall, and F-score results between ESA and econo-ESA were also similar; when the results were different, the gaps were small. The experiments on running cost showed that econo-ESA was faster than ESA. Because the decremental percentage was set to 50%, the procedure was almost twice as fast, as per our expectation.

We found that using a 60% of the index matrix can be faster than 50%. This case also has a better *PCC* than 50% in the 0.6 similarity threshold. Based on these experiments, the 60% index matrix can also be considered as a good candidate for an econo-ESA proposal. For further implementation, we recommend the use of econo50 for long texts and econo60 for short texts.

Our future work on this topic will investigate the decremental patterns of the index matrix. Different choices of documents during the dimensional reduction would have provided different results. A reduction based on topics using Wikipedia categories seems as reasonable as reductions based on clustering or classification techniques.

## Electronic supplementary material

Additional file 1: **MSE of three models.** The MSE file can be opened with MS Excel, MS Excel viewer, or OpenOffice Calc applications. This file contains MSE calculations of three models mentioned in the safe dimensional reduction section. The file shows yellow highlights on model choosing based on the MSE. A logarithmic model is chosen with coefficient values derived from Calc Solver. The file also shows blue highlights in the least squared error, which were used for critical point determinations of Wikipedia’s dimensions decrementation. (XLS 20 KB)
